# Real-world survival outcomes of heavily pretreated patients with refractory HR+, HER2−metastatic breast cancer receiving single-agent chemotherapy—a comparison with MONARCH 1

**DOI:** 10.1007/s10549-020-05838-5

**Published:** 2020-08-12

**Authors:** Hope S. Rugo, Veronique Dieras, Javier Cortes, Debra Patt, Hans Wildiers, Joyce O’Shaughnessy, Esther Zamora, Denise A. Yardley, Gebra Cuyun Carter, Kristin M. Sheffield, Li Li, Valerie A. M. Andre, Xiaohong I. Li, Martin Frenzel, Yu-Jing Huang, Maura N. Dickler, Sara M. Tolaney

**Affiliations:** 1 Comprehensive Cancer Center, University of California San Francisco, San Francisco, CA USA; 2 Centre Eugene Marquis UNICANCER, Rennes Cedex, France; 3 IOB Institute of Oncology, Quironsalud Group, Madrid, Spain; 4 IOB Institute of Oncology, Quironsalud Group, Barcelona, Spain; 5 Vall D’Hebron University Hospital, Vall D’Hebron Institute of Oncology, Barcelona, Spain; 6 Texas Oncology, Austin, TX USA; 7 US Oncology, Dallas, TX USA; 8 Department of General Medical Oncology, University Hospital Gasthuisberg, Leuven, Belgium; 9 Texas Oncology, US Oncology, Baylor University Medical Center, Dallas, TX USA; 10 Sarah Cannon Research Institute, Tennessee Oncology PLLC, Nashville, TN USA; 11 Eli Lilly and Company, Indianapolis, IN USA; 12 Dana-Farber Cancer Institute, Boston, MA USA

**Keywords:** Abemaciclib, Electronic health records, Metastatic breast cancer, Overall survival, Real-world evidence, Retrospective study, Single-arm trial, Real-world control arm

## Abstract

**Purpose:**

In MONARCH 1 (NCT02102490), single-agent abemaciclib demonstrated promising efficacy activity and tolerability in a population of heavily pretreated women with refractory HR+, HER2− metastatic breast cancer (MBC). To help interpret these results and put in clinical context, we compared overall survival (OS) and duration of therapy (DoT) between MONARCH 1 and a real-world single-agent chemotherapy cohort.

**Methods:**

The real-world chemotherapy cohort was created from a Flatiron Health electronic health records-derived database based on key eligibility criteria from MONARCH 1. The chemotherapies included in the cohort were single-agent capecitabine, gemcitabine, eribulin, or vinorelbine. Results were adjusted for baseline demographics and clinical differences using Mahalanobis distance matching (primary analysis) and entropy balancing (sensitivity analysis). OS and DoT were analyzed using the Kaplan–Meier method and Cox proportional hazards regression.

**Results:**

A real-world single-agent chemotherapy cohort (*n* = 281) with eligibility criteria similar to the MONARCH 1 population (*n* = 132) was identified. The MONARCH 1 (*n* = 108) cohort was matched to the real-world chemotherapy cohort (*n* = 108). Median OS was 22.3 months in the abemaciclib arm versus 13.6 months in the matched real-world chemotherapy cohort with an estimated hazard ratio (HR) of 0.54. The median DoT was 4.1 months in MONARCH 1 compared to 2.9 months in the real-world chemotherapy cohort with HR of 0.76.

**Conclusions:**

This study demonstrates an approach to create a real-world chemotherapy cohort suitable to serve as a comparator for trial data. These exploratory results suggest a survival advantage and place the benefit of abemaciclib monotherapy in clinical context.

## Introduction

Hormone receptor positive (HR +), human epidermal growth factor receptor 2 negative (HER2−) is the most prevalent subtype of invasive breast cancer and accounts for approximately 70% of all cases [1]. Metastatic breast cancer (MBC) remains a universally fatal disease, with overall survival (OS) limited to 2 to 3 years on average [2]. Despite the availability of endocrine therapy for the treatment of HR+, HER2− MBC, benefits progressively diminish with the development of resistance and progressive disease (PD).

Inhibitors of cyclin-dependent kinases (CDKs) 4 & 6, in combination with endocrine therapy, have been recommended by the National Comprehensive Cancer Network (NCCN) [3] and the European Society for Medical Oncology (ESMO) for treatment of HR+, HER2− advanced breast cancer (ABC) [4]. After progression on multiple lines of endocrine-based therapy, either alone or in combination with agents such as CDK4 & 6 inhibitors, sequential single-agent cytotoxic chemotherapy becomes the standard of care for most patients [3]. Cytotoxic chemotherapy is associated with substantially more toxicity compared to endocrine therapies [5–8]. The addition of CDK4 & 6 inhibitors to endocrine therapy has markedly improved disease control in both the first and second or greater line settings and recently have been shown to improve survival in several settings [9–13].

Abemaciclib is a potent and selective oral, small-molecule inhibitor of CDK4 & 6 which leads to sustained cell cycle arrest when dosed on a continuous schedule [14]. Abemaciclib has received regulatory approval globally in combination with an aromatase inhibitor as initial endocrine-based therapy for the treatment of postmenopausal women with HR+, HER2− ABC or MBC, in combination with fulvestrant for the treatment of women with HR+, HER2− ABC or MBC with disease progression following endocrine therapy, and in the United States (US) as a monotherapy for the treatment of adult patients with HR+, HER2− ABC or MBC with disease progression following endocrine therapy and prior chemotherapy in the metastatic setting [3, 4, 9]. Abemaciclib has demonstrated clinical activity as monotherapy in patients with HR+, HER2− MBC heavily pretreated with both endocrine and chemotherapy in the metastatic setting in MONARCH 1 (NCT02102490) [15].

MONARCH 1 was a single-arm phase II trial including patients with disease progression on or after endocrine therapy, with prior exposure to a taxane, and with at least 2 prior chemotherapy regimens including at least 1 in the metastatic setting. The objective response rate (ORR) was 19.7% (95% CI [confidence interval], 13.3, 27.5), and the median OS was 22.3 months [15]. Although this was a single-arm study, the ORR and OS observed in MONARCH 1 suggest single-agent abemaciclib may offer a more favorable benefit-risk profile than what might be expected in patients receiving cytotoxic chemotherapy [6, 8]. At the time of the MONARCH 1 trial, treatment options for this patient population were typically limited to chemotherapy [15]. However, without a comparator arm, it is difficult to put these findings into clinical context relative to available treatment options.

Traditionally, historical controls from previous clinical trials have been used to provide context for results from single-arm trials [16, 17], but this approach may be hampered by differing patient populations or lines of treatment. In contrast, real-world data (RWD) allow for selection of a more contemporaneous cohort of patients who match relevant trial criteria and patient-level data, and allow for matching between real-world and trial cohorts to balance patient characteristics [16, 18, 19]. This retrospective cohort study used RWD from patients treated in a clinical practice setting for MBC to create a single-agent chemotherapy control arm to help contextualize the results observed in MONARCH 1.

## Materials and methods

### Data source

This retrospective study utilized the Flatiron Health electronic health records (EHR)-derived database and included patients diagnosed with MBC from 01 January, 2011, to 28 February, 2018. The Flatiron Health database is a US-based longitudinal, demographically and geographically diverse database derived from EHR data from over 280 cancer clinics (~ 800 sites of care) representing more than 2.1 million active cancer patients. As of March 2018, the Flatiron MBC cohort included more than 15,000 patients with MBC from approximately 180 clinics. Patients were included in the cohort if they were stage IV at initial diagnosis or if they developed recurrent MBC after an initial diagnosis of early stage breast cancer. The database includes both structured and some unstructured EHR data elements, such as patient demographics (gender, race, birth year, and state of residence), type of cancer facility visited (community vs. academic), clinical diagnoses, laboratory data, biomarker tests and results, medications ordered and/or administered, line of therapy (derived), month and year of death, and other patient clinical characteristics including cancer stage at initial diagnosis and performance status (PS). Date of death was derived from EHR data, commercial death data, and the Social Security Death Index [20].

### Real-world chemotherapy cohort key inclusion and exclusion criteria

The inclusion and exclusion criteria applied to the Flatiron Health MBC Cohort for this study were intended to recapitulate the MONARCH 1 trial key eligibility criteria [15], where it was feasible to do so, in order to provide the most relevant real-world comparator to the MONARCH 1 patient population (Table 1). The criteria consisted of female patients with a diagnosis of HR+, HER2− MBC who received monotherapy with capecitabine, gemcitabine, eribulin, or vinorelbine in second or later lines of therapy. These agents were selected based on ESMO and NCCN guidelines for single-agent chemotherapy for patients in this setting.

**Table 1:** Inclusion criteria for MONARCH 1 and real-world chemotherapy cohort

Key features	MONARCH 1	Real-world cohort^a^
Indication	MBC	MBC
HR status	Positive	Positive
HER2 status	Negative	Negative
Prior endocrine therapy in adjuvant and/or metastatic setting	Yes	Not required^a^ but prior ET in metastatic disease explored in sensitivity analyses
Number of prior chemotherapy regimens in metastatic setting	1 or 2	1 or 2
ECOG PS	0 or 1	0 or 1
Prior taxane containing regimen in adjuvant or metastatic setting	Yes	Not required^b^
Prior CDK4 & 6 therapy	Not permitted	Not permitted
CNS metastases	Not permitted	Not permitted

*CDK* Cyclin-dependent kinase, *CNS* central nervous system, *ECOG PS* eastern cooperative oncology group performance status, *EHR* electronic health record, *ET* endocrine therapy, *HER2* human epidermal growth factor receptor 2, *HR* hormone receptor, *MBC* metastatic breast cancer

^a^Analyses were conducted both requiring and not requiring (primary analysis) prior endocrine therapy in the metastatic setting

^b^Prior taxane use was ‘not required’ in the real-world chemotherapy cohort because the Flatiron Health EHR database has incomplete data in the adjuvant setting

Patients received at least 1 but no more than 2 lines of chemotherapy for those in the advanced setting prior to receipt of the single-agent chemotherapies listed above. Those included had an Eastern Cooperative Oncology Group (ECOG) performance status of 0 or 1 (within 60 days before or 30 days after the eligible line of therapy). Patients who received CDK4 & 6 inhibitor drugs (palbociclib, ribociclib, and/or abemaciclib) in prior lines of therapy were excluded. Patients who received prior HER2 targeted therapy were excluded. Patients with diagnosis codes for central nervous system (CNS) metastasis (ICD 9: 198.3 and 198.4; ICD 10: C79.31, C79.32, and C79.49) on or before the eligible line of therapy were excluded.

Due to limited information within the data on the adjuvant setting, prior taxane and endocrine therapy in the adjuvant and/or metastatic setting were not required. Patients who received monotherapy with capecitabine, gemcitabine, eribulin, or vinorelbine in 1 or more lines of therapy qualified as eligible based on the above selection criteria. If a patient had only 1 eligible line of therapy, the index drug was the drug contained in the eligible line and the index date was the start date of the eligible line. If the patient had multiple eligible lines of therapy, a line was randomly selected from eligible lines and the index date was the start date of the randomly selected line of therapy (Table 2). The patients were followed from the index date until the date of death, loss to follow-up, or end of the database. The index date must have occurred at least 3 months prior to the end of the database.

**Table 2:** Attrition table for real-world chemotherapy cohort

	*N*
All MBC in Flatiron^a^ Health real-world cohort (Feb 2018)	15,277
Include all female patients with MBC diagnosed from 1 Jan, 2011–28 Feb, 2018	15,073
Include patients who had any single-agent treatment of (capecitabine, gemcitabine, eribulin, or vinorelbine) in line 2 or later	2312
Exclude any patient with prior trastuzumab, pertuzumab, lapatinib, or ado-trastuzumab emtansine treatment	2145
Select patients with eligible lines^b^ (capecitabine, gemcitabine, eribulin, and vinorelbine) without ECOG missing	281

*CDK* Cyclin-dependent kinase, *CNS* central nervous system, *ECOG PS* eastern cooperative oncology group performance status, *EHR* electronic health record, *ER* estrogen receptor, *HER2* human epidermal growth factor receptor 2, *MBC* metastatic breast cancer, *N* total number of patients, *PR* progesterone receptor

^a^Flatiron Health EHR database (https://flatiron.com/real-world-evidence/) 02 2018^*^

^b^Eligible line contains any single-agent treatment (capecitabine, gemcitabine, eribulin, and vinorelbine) (a) received 1–2 lines of therapy containing chemotherapy drug prior to eligible line, (b) had a positive test for ER or positive test for PR on or before the eligible line, (c) had a negative test for HER2 on or before the line containing single-agent treatment, (d) had an ECOG PS ≤ 1 (60 day window prior or 30 days after), (e) no diagnosis codes for CNS metastasis on or before the eligible line, (f) eligible line occurs ≥ 3 months prior to end of database, (g) no CDK4 & 6 inhibitor and no clinical study drug prior to the eligible line

^*^Date of most recent dataset utilized in the analyses, Feb 2018, mortality v2.0

### Statistical analyses

Descriptive statistics were used to summarize baseline demographic and clinical characteristics for the MONARCH 1 and real-world chemotherapy cohorts. The primary endpoint of this exploratory analysis was OS, defined as the time from index date to either censoring or death. Patients without a date of death were censored at the last activity date. OS was analyzed by the Kaplan–Meier method and Cox proportional hazards regression in the matched cohorts. As the primary analysis, the Mahalanobis distance matching method [21] was used to match each patient from MONARCH 1 with a patient from the real-world chemotherapy cohort according to key baseline and disease characteristics. The following characteristics were incorporated into the matching process: age group, race group, number of prior chemotherapies in the metastatic setting, number of prior endocrine therapies in the metastatic setting, prior capecitabine use, and progesterone receptor status. The intent of the matching procedure is to correct for any observed imbalance due to differing baseline demographics and disease characteristics.

As a sensitivity analysis, entropy balancing was performed. Entropy balancing [22] provided a reweighting scheme used to adjust inequalities in distribution of baseline characteristics across the MONARCH 1 and real-world chemotherapy cohorts using pre-specified variables for reweighting. Variables for reweighting using entropy balancing included age group, race group, number of prior chemotherapy regimens in the metastatic setting, number of prior endocrine therapies in the metastatic setting, progesterone receptor status, prior capecitabine use, ECOG PS, and stage at initial diagnosis. The weighted Kaplan–Meier method was applied to the weighted real-world chemotherapy cohort to estimate median OS, and the bootstrap approach was used to estimate 95% confidence interval (CI) of the median OS.

The treatment effect was also evaluated among 2 subsets in additional sensitivity analyses. Since Flatiron Health data are US-based, the first subset was among US patients only, where treatment effect was evaluated between the entire real-world cohort and MONARCH 1 US patients. The second subset was US patients with prior endocrine therapy in the metastatic setting, where treatment effect was evaluated between the real-world cohort with prior endocrine therapy in the metastatic setting and the MONARCH 1 US patients with prior endocrine therapy in the metastatic setting to try and account for the prior endocrine therapy inclusion criterion within the trial.

Duration of therapy was defined as time from index date to last order or administration of the eligible drug during the line of therapy. If the drug was an oral medication, then 30 days were added to the last order date to assume a 30-day supply. Quartiles and median duration of treatment along with 95% CIs were estimated using the Kaplan–Meier method. The Cox model was used to estimate HR. All hypothesis tests were conducted at a 2-sided alpha level of 0.05, unless otherwise stated. All CIs were given at a 2-sided 95% level, unless otherwise stated. Data were analyzed using SAS version 9.2 or later (SAS Institute Inc.) and R (The R Foundation).

## Results

After applying the inclusion and exclusion criteria, 281 patients were identified who received an eligible line of therapy and were included in the real-world chemotherapy cohort (Table 2).

### Baseline demographics and clinical characteristics

Compared to the MONARCH 1 cohort (*n* = 132), the real-world cohort (*n* = 281) was more likely to be 65 years or older (43.4% vs. 31.8%, *p* = 0.03) and less likely to be white (68.3% vs. 93.9%, *p* < 0.0001) (Table 3). A majority of the patients in MONARCH 1 (70 patients, 53.0%) were enrolled at sites in the US. Unlike the real-world chemotherapy cohort which included all US patients, patients in MONARCH 1 were also enrolled at sites in Belgium (28 patients, 21.2%), Spain (23 patients, 17.4%), and France (11 patients, 8.3%). Only 17 (12.9%) patients in the MONARCH 1 cohort had not received prior endocrine therapy in the metastatic setting compared to 114 (40.6%) patients in the real-world chemotherapy cohort. More than half (76, 57.6%) had prior capecitabine in the MONARCH 1 cohort compared to only 73 (26.0%) in the real-world chemotherapy cohort (Table 3). The cohorts were similar with respect to number of prior lines of chemotherapy in the metastatic setting and progesterone receptor status.

**Table 3:** Selected baseline characteristics: before and after Mahalanobis distance matching

Factors	Before matching			After matching		
	MONARCH 1 (*N* = 132)	Real-world cohort (*N* = 281)	*p-*value	MONARCH 1 (*N* = 108)	Real-world cohort (*N* = 108)	*p-*value
Pooled age Group, *n* (%)			0.031			1.0
< 65 years	90 (68.2)	159 (56.6)		72 (66.7)	71 (65.7)	
≥ 65 years	42 (31.8)	122 (43.4)		36 (33.3)	37 (34.3)	
Pooled race group, *n* (%)			< .0001			0.569
Other	8 (6.1)	89 (31.7)		8 (7.4)	5 (4.6)	
White	124 (93.9)	192 (68.3)		100 (92.6)	103 (95.4)	
Lines of chemotherapy^a^, *n* (%)			0.290			0.783
1 Regimen	67 (50.8)	159 (56.6)		61 (56.5)	64 (59.3)	
2 Regimens	65 (49.2)	122 (43.4)		47 (43.5)	44 (40.7)	
Lines of prior ET^a^, *n* (%)			< .0001			0.984
0 Regimen	17 (12.9)	114 (40.6)		17 (15.7)	16 (14.8)	
1 Regimen	48 (36.4)	77 (27.4)		40 (37.0)	42 (38.9)	
2 Regimens	25 (18.9)	54 (19.2)		23 (21.3)	24 (22.2)	
3 + Regimens	42 (31.8)	36 (12.8)		28 (25.9)	26 (24.1)	
PgR Status						0.879
Negative	35 (26.5)	99 (35.2)	.090	29 (26.9)	31 (28.7)	
Positive	95 (72.0)	179 (63.7)		79 (73.1)	77 (71.3)	
Prior capecitabine use, *n* (%)			< .0001			0.586
No	56 (42.4)	208 (74.0)		54 (50.0)	59 (54.6)	
Yes	76 (57.6)	73 (26.0)		54 (50.0)	49 (45.4)	

Fisher’s exact test was used for *p-*value

Patients with missing baseline disease characteristics were removed from the matching protocol

*ET* Endocrine therapy, *N* total number of patients, *n* number of patients within a specific category, *PgR* progesterone receptor

^a^In metastatic setting

The Mahalanobis distance matching method was used to select patients who had the shortest Mahalanobis distances from the 132 patients in MONARCH 1 and the 281 patients in the real-world chemotherapy cohort. Patients with missing baseline disease characteristics were removed from the distance calculation. Following Mahalanobis distance matching, 108 patients from the MONARCH 1 cohort were matched to 108 patients from the real-world chemotherapy cohort, and the cohorts had similar patient and disease characteristics. In the matched cohorts, no statistically significant differences existed in age, race, lines of chemotherapy in the metastatic setting, prior endocrine therapy in the metastatic setting, progesterone receptor status, and prior capecitabine use (Table 3). The prior therapy profile between the matched MONARCH 1 and real-world chemotherapy cohort was also similar (Table 4). In the MONARCH 1 cohort there was greater tamoxifen (29, 26.9% vs. 12, 11.1%), letrozole (40, 37.0% vs. 20, 18.5%), and bevacizumab (13, 12.0% vs. 0) use compared to the real-world chemotherapy cohort in the metastatic setting (Table 4).

**Table 4:** Prior therapy comparison between matched MONARCH 1 and real-world chemotherapy cohort

Patients with any prior therapy	Monarch 1 *N* = 108	Real-world cohort *N* = 108
Chemotherapy^a^, n (%)	108 (100)	108 (100)
Paclitaxel	55 (50.9)	50 (46.3)
Capecitabine	51 (47.2)	49 (45.4)
Docetaxel	18 (16.7)	11 (10.2)
Cyclophosphamide	13 (12.0)	7 (6.5)
Gemcitabine	10 (9.3)	15 (13.9)
Eribulin	6 (5.6)	7 (6.5)
Other^b^	34 (31.5)	34 (31.5)
Endocrine therapy^a^, *n* (%)	91 (84.3)	92 (85.2)
Fulvestrant	52 (48.1)	59 (54.6)
Exemestane	45 (41.7)	42 (38.9)
Letrozole	40 (37.0)	20 (18.5)
Tamoxifen	29 (26.9)	12 (11.1)
Anastrozole	19 (17.6)	22 (20.4)
Other^c^	14 (13.0)	10 (9.3)
Targeted therapy^a^, *n* (%)	48 (44.4)	32 (29.6)
Everolimus	29 (26.9)	32 (29.6)
Bevacizumab	13 (12.0)	0
Other^d^	13 (12.0)	1 (0.9)
Other^a^, *n* (%)	21 (19.4)	2 (1.9)
Investigational drug	14 (13.0)	0
Other^e^	10 (9.3)	2 (1.9)

*N* Total number of patients, *n* number of patients within a specific category

^a^Any single therapy with > 10% in either arm is listed; all other therapies, except eribulin (included in the table) are combined into ‘other’ in each category

^b^Other chemotherapy agents include doxorubicin, carboplatin, fluorouracil, vinorelbine, epirubicin, cyclophosphamide w/epirubicin hydrochloride/f, cyclophosphamide w/doxorubicin, methotrexate, mitoxantrone, oxaliplatin, paclitaxel w/carboplatin, cisplatin, doxorubicin pegylated liposomal, and ixabepilone

^c^Other endocrine therapy regimens include megestrol, abiraterone, bicalutamide, diethylstilbestrol, enzalutamide, gonadorelin, goserelin, leuprorelin, medroxyprogesterone, toremifene, triptorelin, and leuprolide

^d^Other targeted therapies include dasatinib, ganetespib, taselisib, abexinostat, buparlisib, olaparib, ramucirumab, ridaforolimus, ruxolitinib, veliparib, and cetuximab

^e^Other other therapies include denosumab, zoledronic acid, fluoxymesterone, prednisolone, leucovorin, and sorafenib

### Overall survival

**Fig. 1:**
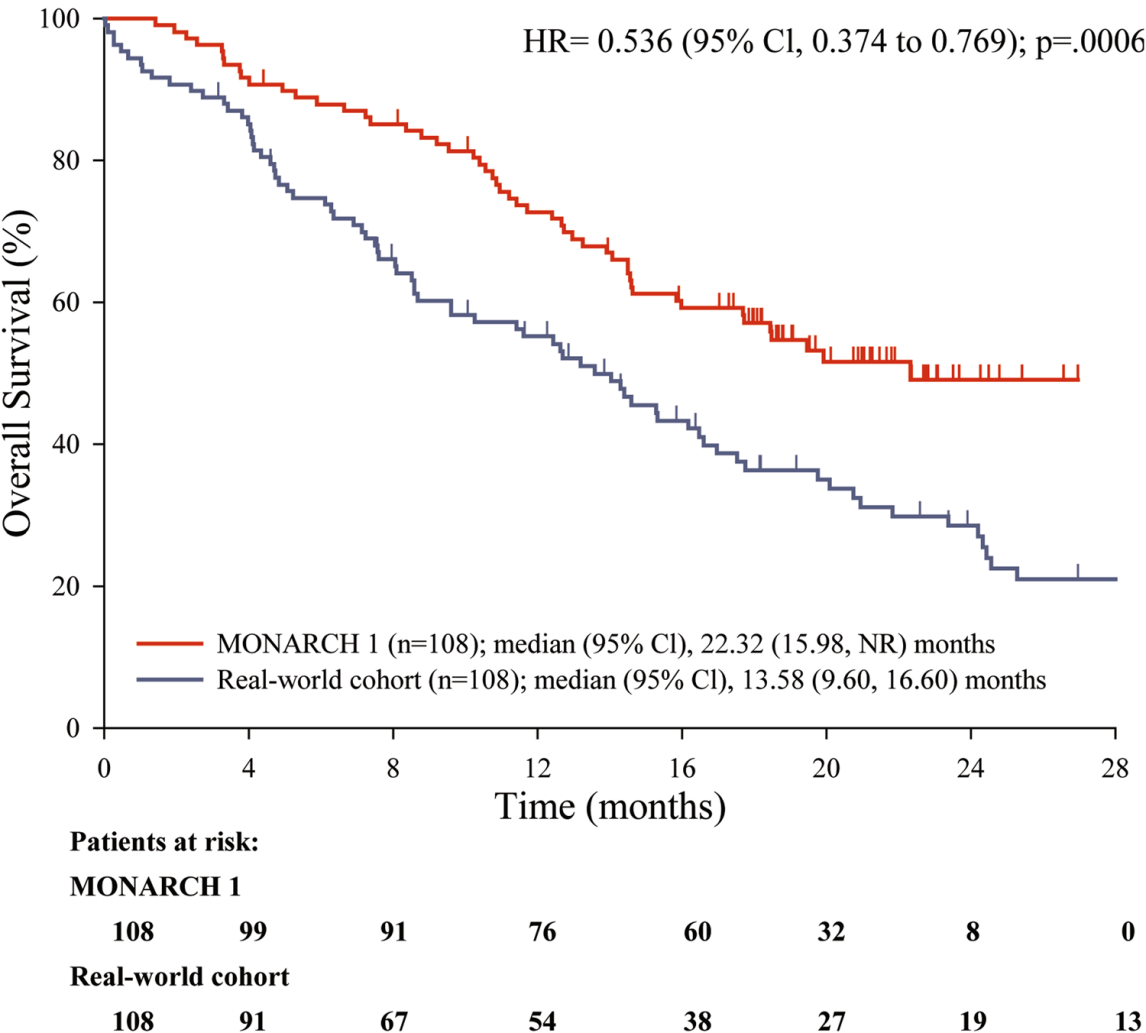
Overall survival between matched MONARCH 1 and real-world chemotherapy cohort. *p-*value (2-sided)—LOGRANK unstratified for comparing MONARCH 1 with real-world chemotherapy cohort. *CI* Confidence interval, *HR* hazard ratio, *NR *not recorded

Using the matching method, the adjusted median OS was 22.3 months (95% CI: 16.0, NR) in MONARCH 1 and 13.6 months (95% CI: 9.6, 16.6) in the real-world chemotherapy cohort. The estimated HR between the 2 matched adjusted groups was 0.536 (95% CI: 0.37, 0.77) (Table 5, Fig. 1).

**Table 5:** Overall survival between matched MONARCH 1 and real-world chemotherapy cohort

	Monarch 1 *N* = 108	Real-world cohort *N* = 108	Difference/*p*-value
Number of deaths, *n* (%)	50 (46.3)	79 (73.1)	
Patients censored, *n* (%)	58 (53.7)	29 (26.9)	
No documented deaths	58 (53.7)	29 (26.9)	
Survival rate, % (95% CI)^a^			
4-month	91.7 (84.6, 95.6)	85.1 (76.9, 90.6)	6.5 (-2.0, 15.0)/*p* = 0.133
8-month	85.1 (76.9, 90.6)	66.1 (56.2, 74.2)	19.0 (7.8, 30.3)/*p* = 0.0009
12-month	72.7 (63.2, 80.2)	55.2 (45.1, 64.1)	17.5 (4.8, 30.3)/*p* = 0.007
24-month	49.1 (38.1, 59.2)	28.5 (19.5, 38.1)	20.6 (6.4, 34.8)/*p* = 0.004

Overall survival rates were estimated using the Kaplan–Meier method. Corresponding 95% CIs were estimated using the methods of Brookmeyer and Crowley, and Greenwood, respectively

*CI* Confidence interval, *N* total number of patients, *n* number of patients within a specific category

^a^95% CIs and 2-sided *p*-values for the difference between rates were calculated based on normal approximation

**Fig. 2:**
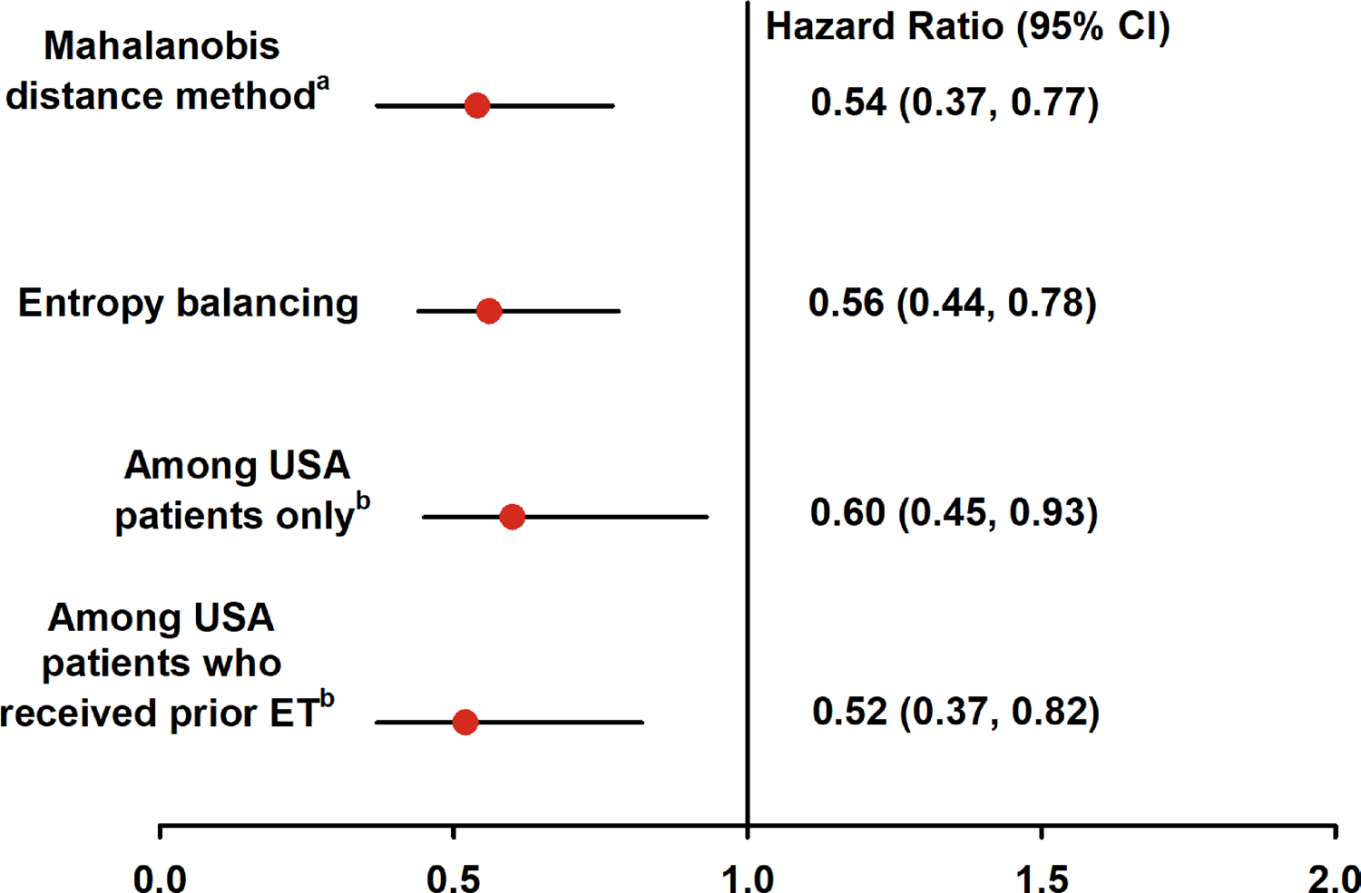
Sensitivity analyses of overall survival. ^a^Method in main analysis. ^b^Entropy balancing method was applied. Sample sizes are as follows: Mahalanobis distance method: real-world chemotherapy cohort (*n* = 108) vs. MONARCH 1 (*n* = 108). Entropy balancing: real-world chemotherapy cohort (*n* = 281) vs. MONARCH 1 (*n* = 132). US patients only: real-world chemotherapy cohort (*n* = 281) vs. US patients only (n = 70). US patients who received prior ET: real-world chemotherapy cohort with prior ET (n = 167) vs. US patients who received prior ET in metastatic setting (n = 62). Abbreviations: *ET* endocrine therapy

The results of sensitivity analyses, using entropy balancing as a secondary analytic approach, were consistent with the Mahalanobis distance matching results. The adjusted median OS was 12.7 months in the real-world cohort (*n* = 281) with a HR of 0.56 (95% CI from bootstrapping: 0.44, 0.78). The sensitivity analysis in the real-world chemotherapy cohort (*n* = 281) and MONARCH 1 US patients only (*n* = 70) was consistent with a HR of 0.60 (95% CI: 0.45, 0.93). The sensitivity analysis of the patients with prior endocrine therapy for MBC in the real-world chemotherapy cohort (*n* = 167) and in the MONARCH 1 US cohort (*n* = 62) was also consistent with a HR of 0.52 (95% CI: 0.37, 0.82) (Fig. 2).

### Duration of therapy

Duration of therapy was not significantly different between the 2 cohorts. The median duration of therapy was 4.1 months in MONARCH 1 compared to 2.9 months in the real-world chemotherapy cohort (*p* = 0.050) with a HR of 0.764 (95% CI: 0.58, 1.00) (Table 6).

**Table 6:** Duration of therapy

Duration of therapy after mahalanobis distance matching	MONARCH 1 *N* = 108	Real-world cohort *N* = 108	Difference*/p-value*
Number of events, *n* (%)	103 (95.4)	104 (96.3)	
Discontinued	103 (95.4)	104 (96.3)	
Patients censored, *n* (%)	5 (4.6)	4 (3.7)	
No documented discontinuation	5 (4.6)	4 (3.7)	
Minimum^a^, months	0.43	0.03	
25^th^ percentile (95% CI)	1.87 (1.87, 2.24)	1.45 (0.95, 2.07)	
Median (95% CI)	4.08 (2.79, 5.52)	2.89 (2.33, 3.98)	
75^th^ percentile (95% CI)	8.28 (6.02, 11.54)	6.67 (4.83, 9.99)	
Maximum, months	26.56 +	26.33	
*p*-value (2-sided)—log rank unstratified			*p* = 0.05
Hazard ratio (95% CI)—unstratified			0.764 (0.581, 1.004)

Quartiles and duration of treatment survival rates, along with 95% CIs were estimated using the Kaplan–Meier method. Corresponding 95% CIs were estimated using the methods of Brookmeyer and Crowley, and Greenwood, respectively

*CI* Confidence interval, *N* total number of patients, *n* number of patients within a specific category

^a^For minimum/maximum, ‘ + ’ indicates a censored observation

### Post-discontinuation therapy

Post-discontinuation therapy was consistent between the 2 cohorts. In total, 71 patients in the real-world chemotherapy cohort received at least 1 subsequent therapy compared with 77 patients in MONARCH 1. A total of 61 patients in the real-world cohort received chemotherapy as any post-discontinuation therapy compared with 65 patients in MONARCH 1. However, more patients in the real-world chemotherapy cohort received palbociclib (*n* = 32) or endocrine therapy (*n* = 50) versus patients in MONARCH 1 (*n* = 0 receiving palbociclib, *n* = 13 receiving endocrine therapy) as any post-discontinuation therapy (Table 7).

**Table 7:** Summary of post-discontinuation treatment

Any post-discontinuation therapy after mahalanobis distance matching	MONARCH 1 *N* = 108	Real-world cohort *N* = 108
Patients with any post-discontinuation therapy, *n* (%)	77 (71.3)	71 (65.7)
Chemotherapy^a^, *n* (%)	65 (60.2)	61 (56.5)
Capecitabine	26 (24.1)	14 (13.0)
Eribulin	18 (16.7)	18 (16.7)
Doxorubicin	16 (14.8)	3 (2.8)
Paclitaxel	15 (13.9)	16 (14.8)
Vinorelbine	13 (12.0	11 (10.2)
Gemcitabine	3 (2.8)	17 (15.7)
Doxorubicin pegylated liposomal	0	19 (17.6)
Other^b^	18 (16.7)	50 (46.3)
Endocrine therapy^c^, *n* (%)	13 (12.0)	50^d^ (46.3)
Fulvestrant	6 (5.6)	17 (15.7)
Exemestane	3 (2.8)	15 (13.9)
Letrozole	3 (2.8)	26 (24.1)
Other^e^	4 (3.7)	9 (8.3)
Targeted therapy, *n* (%)	8 (7.4)	41 (38.0)
Everolimus	5 (4.6)	10 (9.3)
Palbociclib	0	32 (29.6)
Other^f^	3 (2.8)	5 (4.6)
Other, *n* (%)	8 (7.4)	6 (5.6)
Investigational drug	6 (5.6)	4 (3.7)
Other^g^	2 (1.9)	2 (1.9)

*CDK* Cyclin-dependent kinases, *N* total number of patients, *n* number of patients within a specific category

^a^Any single chemotherapy agent with > 10% in either arm is listed; all other therapies are combined into ‘other’

^b^Other chemotherapy agents include cyclophosphamide, fluorouracil, docetaxel, cisplatin w/fluorouracil, cyclophosphamide w/epirubicin hydrochloride/f, cyclophosphamide w/fluorouracil/methotrexate, epirubicin, lurbinectedin, methotrexate, thiotepa, carboplatin, cisplatin, etoposide, irinotecan, and ixabepilone

^c^Any endocrine, targeted, or other therapy with > 5% in either arm is listed; all other therapies are combined into ‘other’

^d^32 out of 50 patients who received endocrine therapy also received concurrent CDK4 & 6 inhibitor

^e^Other endocrine therapy regimens include tamoxifen, orteronel, anastrozole, and leuprolide

^f^Other targeted therapies include bevacizumab, cabozantinib, trastuzumab, nivolumab, and ribociclib

^g^Other therapies include dexrazoxane, doxycycline, and leucovorin

## Discussion

Abemaciclib has demonstrated clinical activity as a monotherapy in patients with HR+, HER2− MBC heavily pretreated in the advanced setting with both endocrine and chemotherapy in MONARCH 1 (NCT02102490) [15]. Here, we compared OS and duration of treatment in MONARCH 1 with a matched real-world cohort of patients who received standard-of-care treatment with single-agent chemotherapy and had not previously received CDK4 & 6 inhibitors using the Flatiron database. Although MONARCH 1 did not include a control arm, this real-world chemotherapy cohort provided a data source representative of a control arm. The OS was significantly longer in the MONARCH 1 cohort at 22.3 months compared to 13.6 months in the matched real-world chemotherapy cohort. Duration of treatment was not significantly different between the two groups; however, it was numerically longer in the MONARCH 1 cohort (4.1 months) compared to the real-world cohort (2.9 months). Altogether, these results suggest a possible survival advantage in favor of abemaciclib as a monotherapy in these patients.

In certain cases, regulatory agencies have accepted the use of real-world control arms to contextualize results from single-arm trials to support regulatory decisions. The increasing accessibility of digital health data, in combination with rising costs and recognized limits of traditional trials, has renewed interest in the use of RWD to enhance the efficiency of research and bridge the evidentiary gap between clinical research and practice [23]. The FDA Real-World Evidence (RWE) Framework notes RWE can be used as the basis for external controls in some situations [16]. Furthermore, RWE has been pivotal in some European regulatory decisions involving conditions with significant unmet need and when a randomized clinical trial is unfeasible or unethical [24]. For example, in the case of blinatumomab, the real-world cohort was helpful in supporting accelerated approval for the treatment of acute lymphoblastic leukemia by the European Medicines Agency [25, 26]. In the case of avelumab as a monotherapy for metastatic Merkel cell carcinoma (mMCC), RWE was used to characterize the natural history of mMCC and was offered to regulators as a benchmark. A subset of trial patients who responded well to treatment was identified and the benefit documented through contrast with the RWE benchmark data, leading to regulatory approval in the US, European Union, and Japan for that subset [19, 27, 28].

There have been improvements in the quality of data over the past several years, with access to recent data containing rich clinical variables and relevant endpoints. For example, the Flatiron Health EHR-derived database represents a diverse group of community cancer clinics, ranging from small practices to large multicenter practices. A full copy of each patient’s medical record is pulled into a central repository for processing. Structured data such as demographics, medications, and routine laboratory tests are harmonized and normalized to a standard ontology and common data model. These structured data are processed and harmonized centrally by Flatiron’s technology-assisted data engine and made accessible for research and analytics. Unstructured data such as case notes, pathology reports, and complex laboratory tests are turned into discrete analyzable data using technology driven abstraction [29, 30].

We attempted to mimic the eligibility criteria for MONARCH 1 in the Flatiron cohort, and we used matching and balancing methods to control for differences in key measured confounders between cohorts. To adjust for potential differences, the Mahalanobis distance matching method [21] was used to match each patient from MONARCH 1 with a patient from the real-world chemotherapy cohort. The aim of this method was to select a subset of patients from the real-world chemotherapy cohort with the most comparable baseline and disease characteristic to the MONARCH 1 population. Following this procedure, known and measured baseline characteristics were balanced between the cohorts. Any observed imbalance due to differing baseline and disease characteristics was corrected, as illustrated by the lack of significance in any of the *p*-values.

Some of the potential limitations to this approach have been addressed through sensitivity analyses. However, we were unable to match the trial eligibility criteria exactly due to a lack of data availability in Flatiron (such as incomplete data on adjuvant therapy) and unmeasured confounders. Data on adjuvant therapy, including prior taxane use, are not complete in the real-world chemotherapy cohort because patients may have received care prior to adoption of an EHR at the practice, or patients may have received care at a practice outside the Flatiron Health network. Other variables such as sites of metastatic disease (e.g. visceral disease or liver metastases), previous cancers, and comorbidities were not included in the Flatiron Health data. Therefore, residual differences between the real-world chemotherapy and MONARCH 1 cohorts may have contributed to the observed outcomes, even after matching on key measured variables. Another limitation is orders for oral therapies may be incomplete in the structured EHR data, as subsequent refills may not be documented, thus duration of treatment may have been underestimated in the real-world chemotherapy cohort. Another limitation inherent to real-world studies is the potential for selection bias. The fact that clinicians chose to give 1 cohort of patients’ chemotherapy instead of endocrine or targeted therapy could imply patients in the chemotherapy only arm is a higher risk group of patients.

Furthermore, the MONARCH 1 and real-world chemotherapy cohort were not contemporaneous. MONARCH 1 enrolled patients from June 2014 through April 2015, while the real-world chemotherapy cohort included patients with index dates from January 2011 through February 2018. As a result, 29.6% of patients in the real-world chemotherapy cohort received a CDK4 & 6 inhibitor following discontinuation of the index therapy. This suggests the survival in the real-world cohort is potentially longer than it would have been in a truly contemporaneous cohort, thus the difference in overall survival observed in this analysis may be underestimated. Finally, MONARCH 1 included patients from Europe and the US, while the real-world chemotherapy cohort was from the US only. However, sensitivity analyses suggest results are consistent in the subset of MONARCH 1 patients from the US (Fig. 2).

## Overall conclusion

Methodological advances in statistical analyses and improvements in data quality enable the use of a real-world single-agent chemotherapy cohort as an external comparator arm. This study demonstrated an approach to create a real-world chemotherapy cohort to serve as a suitable comparator for MONARCH 1. These exploratory results suggest a possible survival advantage in heavily pretreated patients with advanced MBC treated with abemaciclib monotherapy compared to those treated with single-agent chemotherapy.

## Data Availability

Eli Lilly and Company provides access to all individual participant data collected during the trial, after anonymization, with the exception of pharmacokinetic or genetic data. Data are available to request 6 months after the indication studied has been approved in the United States and European Union and after primary publication acceptance, whichever is later. No expiration date of data requests is currently set once data are made available. Access is provided after a proposal has been approved by an independent review committee identified for this purpose and after receipt of a signed data sharing agreement. Data and documents (including the study protocol, statistical analysis plan, clinical study report, and blank or annotated case report forms) will be provided in a secure data sharing environment. For details on submitting a request, see the instructions provided at www.vivli.org.
